# Using Preoperative Therapeutic Plasma Exchange in Lupus Anticoagulant Hypoprothrombinemia Syndrome to Achieve Hemostatic Factor II Activity: A Case Report

**DOI:** 10.1002/jca.70150

**Published:** 2026-07-06

**Authors:** Puja Panwar, Sean G. Yates, Rebecca Yarborough, Yu‐Min Shen

**Affiliations:** ^1^ UT Southwestern Medical Center Dallas Texas USA; ^2^ US Oncology Dallas Texas USA

## Abstract

Lupus anticoagulant hypoprothrombinemia syndrome (LA‐HPS) is a rare cause of acquired factor II deficiency associated with lupus anticoagulant due to antiprothrombin antibody, which predisposes to bleeding rather than thrombosis. We report a case of a 48‐year‐old female with LA‐HPS resulting in a bleeding diathesis due to acquired prothrombin or factor II (FII) deficiency in the context of very high titers of anti‐phosphatidylserine/prothrombin antibodies (anti‐PS/PT). In preparation for elective mitral valve replacement surgery due to infective endocarditis, she received four sessions of therapeutic plasma exchange (TPE) preoperatively, followed by IVIG (2 g/kg total in divided doses). Her FII activity improved from 7% to 53% on the morning of her surgery, and her IgM anti‐PS/PT decreased from greater than 150 units to a nadir of 26.4 units. She completed the surgery without bleeding complications. She has since been maintained on immunosuppression with mycophenolate and without bleeding. We illustrate one of the few reported successful uses of TPE to effectively remove the anti‐PS/PT antibodies and optimizing hemostasis by increasing FII activity before a surgical procedure to mitigate the high bleeding risk. We also add to the limited existing evidence that immunosuppression is a reasonable long‐term treatment in patients with anti‐prothrombin antibodies causing severe hypoprothrombinemia with bleeding.

## 1 Introduction

We present a case of a 48‐year‐old female with a history of rheumatic heart disease who developed lupus anticoagulant hypoprothrombinemia syndrome (LA‐HPS), resulting in a bleeding diathesis [1, 2, 3].

## 2 Case Presentation

Before transferring her care to our institution, she suffered from recurrent rectal bleeding and heavy menstrual bleeding that necessitated transfusions and laparoscopic hysterectomy. Due to transfusion needs, a PICC line was placed, which was complicated by mitral valve endocarditis. Her care was then transferred to our institution for further workup and management in preparation for high‐risk mitral valve replacement (MVR) surgery.

At our institution, hemostasis workup showed a prolonged activated partial thromboplastin time (aPTT) of 47 s, prolonged prothrombin time (PT) of 18 s, positive lupus anticoagulant, elevated anticardiolipin IgM (99 MPL; reference range: ≤ 40 MPL), elevated anti‐beta2‐glycoprotein IgM (138 SMU; reference range: ≤ 18.4 SMU), and significantly elevated anti‐phosphatidylserine/prothrombin antibodies (anti‐PS/PT) IgG and IgM both higher than the upper limit of normal reference range > 150 units. Factor IX, VIII, VII, X, and V activities were normal. In the mixing study, the PTT clotting time was significantly prolonged (immediate time 60 s) compared to the patient's baseline PTT of 47 s (PTT reference range: 23–32.5 s), whereas her PT corrected—immediate time 12 s compared to baseline 18 s (PT reference range: 9.5–12.8 s). Prothrombin (factor II) was found to be < 5% (reference range: 80%–129%), thus leading to a diagnosis of LA‐HPS [2, 4].

Before surgery, to lower the anti‐PS/PT antibodies and reduce her bleeding symptoms, she received four weekly Rituximab 375 mg/m^2^ infusions, which helped reduce her GI bleeding and initially decreased the anti‐PS/PT IgM antibody titer to 83.5 units and improved FII activity to a peak of 13% before planned admission for MVR surgery [5]. Unfortunately, this initial response was short‐lived. Prednisone was not used with the underlying infective endocarditis and concern for delayed wound healing after surgery. Given the necessity of the MVR, a multidisciplinary discussion with hematology, transfusion medicine, and thoracic surgery led to the use of therapeutic plasma exchange (TPE) to reduce the anti‐PS/PT antibody titer to increase FII activity.

**FIGURE 1:**
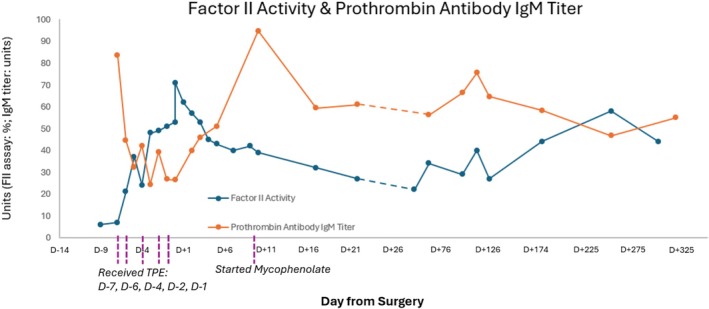
The graph in blue tracks the patient's FII %, while the graph in orange shows the patient's corresponding IgM anti‐PS/PT antibody titer. Day 0 is the date of MVR surgery. Before her hospital admission, the patient's baseline FII activity was mostly < 10%. FII % increased with a concomitant decrease in IgM anti‐PS/PT antibody titers during TPE therapy in the preoperative period. She received TPE on: Day‐7, Day‐6, Day‐4, Day‐2, and Day‐1 to keep factor II activity > 30% preop. The dashed segments indicate temporal extrapolation between non‐adjacent data points.

Upon admission for her planned MVR, her baseline FII activity was 7%, which improved to 21% after her first TPE session (1.2 volume TPE using 2/3 fresh frozen plasma and 1/3 5% albumin as replacement fluid). At our institution, TPE for coagulation factor repletion is typically performed using 2/3 plasma and 1/3 albumin, to balance effective factor replacement with judicious plasma utilization. In this time‐limited pre‐operative setting, we chose an exchange volume of 1.2 to enhance immediate intravascular removal of prothrombin‐directed antibodies and immune complexes compared to 1.0 plasma volume, while plasma replacement directly restored prothrombin and other clotting factors to support surgical hemostasis. FII activity was assessed daily after TPE. After four sessions of TPE, her IgM anti‐PS/PT level decreased to a nadir of 26.4 while the IgG titer remained high (Figure 1). Correspondingly, FII activity increased to greater than 50% (over our goal of 30%) on the morning of the MVR surgical date. MVR proceeded without complications. She remained hemostatically stable with no postoperative bleeding complications. Mycophenolate was started for immunosuppression, and she maintained a FII activity of > 25%. She was also able to undergo abdominal hernia repair 9 months later without additional interventions and had no bleeding complications.

## 3 Discussion

To the best of our knowledge and literature review, this case represents one of only a few reported cases of using preoperative TPE in lupus anticoagulant hypoprothrombinemia syndrome [6, 7]. We showed that the patient's IgM anti‐PS/PT antibody was reduced successfully, and FII activity increased to the hemostatic range before major cardiac surgery despite persistently elevated IgG anti‐PS/PT antibodies.

There are limited case reports of hypoprothrombinemia with bleeding in the preoperative management of patients with LA‐HPS. One case report described a patient with LA‐HPS who successfully underwent complex dental surgery without hemorrhagic complications after receiving rituximab, steroids, and TPE preoperatively [7]. Another case reported in 1987 discussed a 66‐year‐old man with LAHS who was successfully treated with cyclophosphamide and prednisone to correct FII deficiency before elective major surgery [8].

Our patient's IgG anti‐PS/PT antibody remains > 150. This is not uncommonly observed and is likely due to the intravascular vs. extravascular distribution of IgG vs. IgM [9, 10]. The IgM decreased modestly with the initial rituximab course preceding her MVR, reaching a nadir during TPE therapy, and subsequently remained at intermediate titer levels, in the range of 40–60 s with mycophenolate [5]. Fortunately, the reduction in the IgM anti‐PS/PT antibody correlated with the improvement of FII activity. She has tolerated significant hemostatic challenges, including cardiac surgery and abdominal hernia surgery, without bleeding complications.

This case shows the utility of TPE in successfully removing the IgM anti‐PS/PT antibodies and subsequently increasing FII activities to hemostatic levels prior to a necessary and high‐bleeding‐risk surgical procedure.

## Funding

The authors have nothing to report.

## Conflicts of Interest

The authors declare no conflicts of interest.

## Data Availability

Data sharing not applicable to this article as no datasets were generated or analyzed during the current study.
